# Mulching impact of *Jatropha curcas* L. leaves on soil fertility and yield of wheat under water stress

**DOI:** 10.1038/s41598-022-13005-7

**Published:** 2022-05-25

**Authors:** Muhammad Irshad, Faizan Ullah, Sultan Mehmood, Asma A. Al-Huqail, Shah Fahad, Manzer H. Siddiqui, Hayssam M. Ali, Shah Saud, Subhan Danish, Rahul Datta, Khadim Dawar

**Affiliations:** 1 Department of Botany, University of Science and Technology, Bannu, Pakistan; 2 Chair of Climate Change, Environmental Development and Vegetation Cover, Department of Botany and Microbiology, College of Science, King Saud University, Riyadh, 11451 Saudi Arabia; 3 Hainan Key Laboratory for Sustainable Utilization of Tropical Bio Resource, College of Tropical Crops, Hainan University, Haikou, 570228 Hainan China; 4 Department of Agronomy, The University of Haripur, Haripur, 22620 Khyber Pakhtunkhwa Pakistan; 5 College of Life Science, Linyi University, Linyi, 276000 Shandong China; 6 Department of Soil Science, Faculty of Agricultural Sciences and Technology, Bahauddin Zakariya University, Multan, 60800 Pakistan; 7 Department of Geology and Pedology, Faculty of Forestry and Wood Technology, Mendel University in Brno, 61300 Brno, Czech Republic; 8 Department of Soil and Environmental Science, The University of Agriculture, Peshawar, Pakistan

**Keywords:** Ecology, Plant sciences

## Abstract

In present studies we have evaluated mulching impact of *Jatropha curcas* leaves on soil health and yield of two wheat (*Triticum aestivum* L.) varieties Wadan-2017 (rainfed) and Pirsabak-2013 (irrigated) under imposed water stress. Mulch of Jatropha leaves was spread on the soil surface at the rate of 0, 1, 3 and 5 Mg ha^−1^ after seed germination of wheat. Water stress was imposed by skipping irrigations for one month at anthesis stage of wheat maintaining 40% soil field capacity. We found a significant decline in soil microbial biomass carbon (30.27%), total nitrogen (22.28%) and organic matter content (21.73%) due to imposed water stress in non-mulch plots. However, mulch application at 5 Mg ha^−1^ significantly improved soil organic matter (38.18%), total nitrogen (37.75%), phenolics content (16.95 mg gallic acid equivalents/g) and soil microbial biomass carbon (26.66%) as compared to non-mulch control. Soil health indicators like soil carbonates, bicarbonates, electrical conductivity, chloride ions and total dissolved salts were decreased by 5 Mg ha^−1^ mulch application. We noted a decline in yield indicators like spike weight (14.74%), grain spike^−1^ (7.02%), grain length (3.79%), grain width (3.16%), 1000 grains weight (6.10%), Awn length (9.21%), straw weight (23.53%) and total grain yield (5.98%) of wheat due to imposed water stress. Reduction in yield traits of wheat due to water stress was higher in Pirsabak-2013 than Wadan-2017. Jatropha leaves mulch application at 5 Mg ha^−1^ significantly minimized the loss in yield traits of wheat crop caused by water stress. *Jatropha curcas* leaves mulch application at 5 Mg ha^−1^ is recommended for the successful establishment of wheat crop under water deficit conditions.

## Introduction

Due to climate change, in arid and semiarid regions of the world main cause of moisture loss is evaporation from the root zone leading to the creation of water stress^1^. Therefore, in water stress condition plants need more water for their survival. Due to susceptibility of agricultural ecosystem to water stress food safety is threatened throughout the world. Therefore, scientists across the globe are trying to develop technologies for reducing impact of low soil moisture availability on growth and yield of economically important plants^2^. Mulching is one of such techniques found helpful in improving soil fertility and conservation of soil moisture^3^. Mulching also suppresses the weed influx^4^, improves soil texture^5^ and reduces soil erosion^6^. Mulch considerably increases potassium, phosphorus, nitrogen and organic carbon content of soil^7^. Use of organic amendments significantly decreases the toxic effects of heavy metals^8^, minimizes the salinity and sodicity stress effects on crops^9^.

Mulching increases plant productivity and conserves soil moisture content^10^. Mulching technique significantly reduces evaporation by modifying soil surface conditions. Due to mulching more water is available for plant use^11^. Organic mulch increased sesame grain yield from 147 to 250% compared to no-mulching^12^. Similarly rice straw mulch increased twice potato yield^13^. It was reported that yield of beans was increased 33% by mulch^14^. Straw mulch increased root yield of sugar beet from 9.40 to 11.20% and yield of sugar from 8 to 11.30%^15^.

*Jatropha curcas* is oil producing plant and belongs to family Euphorbiaceae. The seeds of Jatropha contain fibers, proteins and oil^16^. Jatropha is a drought-resistant plant species cultivated for controlling soil erosion and non-edible seed oil^17,18^. From seed oil of Jatropha varnish, medicine, lubricants, soap and biodiesel are manufactured^19,20^. Due to its industrial value plantation of Jatropha has been increased in tropical and sub-tropical regions of the world. Jatropha is a deciduous plant species and produces a lot of green matter which can be utilized as a source of mulch for increasing soil fertility. Jatropha leaf has been found as a rich source of minerals like Ca, Mg, K, Zn, Fe and organic matter^21^. However, *J. curcas* plantation may influence soil properties and soil microbial activities due to decomposition of its litter^22^. Therefore, we conducted these studies for evaluating Jatropha leaf mulch effects on soil fertility indicators and yield of wheat.

Wheat (*Triticum aestivum* L.) is an important food crop and can be cultivated in different environments. However, in arid regions water stress is the main ecological factor limiting the productivity of wheat crop throughout the world^23^. It is therefore needed of the day to protect wheat crop from water stress by using green, economical and sustainable technologies. Studies of many researchers like^24–26^ have reported that various mulching techniques increased grain yield of wheat by conserving soil water and improving soil microbial activity. Mineral phosphorus and organic amendments considerably increased soil fertility and yield of wheat^27^. In present studies we have evaluated mulching impact of *Jatropha curcas* leaves on soil health and yield of two wheat varieties Wadan-2017 (rainfed) and Pirsabak-2013 (irrigated) under imposed water stress.

## Materials and methods

### Preparation of organic mulch

Fresh leaves of *Jatropha curcas* were collected from 30 healthy plants (3–5 m tall) and dried in shade. The dried leaves were broken down and then sieved with the help of 2 mm mesh^28^.

### Assessment of organic mulch potential on yield of wheat

In this study certified grains of two wheat (*Triticum aestivum* L*.*) varieties Wadan-2017 (rainfed) and Pirsabak-2013 (irrigated) were used. Details about pedigree and parentage of both the varieties are given in Table 1. Standardized size seeds of wheat were sterilized with 95% ethanol and washed with distilled water. In wheat growing season seeds were sown under natural condition during the years of 2018–2019 and 2019–2020 in District Bannu KP, Pakistan. During wheat growing season of 2018–2019 total rainfall was 284.7 mm while in 2019–2020 it was 227.4 mm. In 2018–2019 minimum and maximum average temperature was 11.02 and 24.07 °C correspondingly. Likewise during wheat growing season of 2019–2020 the average monthly minimum and maximum temperature were 11.19 and 23.29 °C respectively. Before sowing of seeds in the field recommended dose of potassium (50 kg ha^−1^), phosphorus (60 kg ha^−1^) and nitrogen (100 kg ha^−1^) were used. The split plot design was used for this experiment. Main treatments were control (irrigated having 100% soil field capacity) and water stress having (40% soil field capacity). Water stress was imposed by skipping irrigation for one month at anthesis stage of wheat. Application of irrigation during wheat growing season of 2018–2019 are given in Table 2. The plot size was 1 × 1 m^2^, having three replicates per treatment. The seeds were sown 10 cm apart and 5 cm deep in soil. Mulch of Jatropha leaves was spread on the soil surface at the rate of 0, 1, 3 and 5 Mg ha^−1^ after germination of wheat^29^.

**Table 1:** Pedigree/parentage of wheat varieties used in the study.

Variety	Pedigree/parentage	Province of release	Year of release	Moisture regime
Pirsabak-2013	CMSS97M04005T-040Y-020Y-030M-020Y-040M-28Y-3M0Y CS/TH.SC//3*PVN/3/MIRLO/BUC/4/MILAN/5/TILHI	*Khyber Pakhtunkhwa*	2013	Irrigated
Wadan-2017	CMSA04Y00649S-028CRE1Y-010M-3SY-03CRE1Y-010M-03Y-0B YAV79//DACK/RABI/3/SNIPE/4/AE.SQUARROSA	*Khyber Pakhtunkhwa*	2017	Rainfed

**Table 2:** Application of irrigation during 2018–2019 wheat growing season.

Irrigation	Start	End	Duration of days
1	12 November 2018	02 December 2018	20
2	07 December 2018	01 January 2019	25
3	05 January 2019	02 February 2019	28
4	07 February 2019	09 March 2019	30
5	12 March 2019	5 April 2019	24

When the seeds of wheat become fully matured and plants turned yellow, then they were harvested and yield parameters were determined. Thousand seed weight, spike weight, straw weight and yield per plot were determined with digital balance. Straw weight and grain yield of wheat was converted into kg/ha. By digital Vernier Caliper length and width of wheat seeds were determined. Number of grains per spike was counted in each treatment. Awn length of wheat spike was measured with the help of common measuring scale.

### Assessment of organic mulch on soil fertility

After crop harvest soil samples were collected and effect of mulch and water stress on soil fertility was determined. Soil E.C and pH was determined according to standard methods of Richards and Mclean^30,31^. Carbonates, bicarbonates, total dissolved salts, chloride ion, calcium and magnesium were analyzed in soil by the method of Richards^30^. Soil organic matter was determined by the method of Walkley^32^. By the method of Singleton et al.^33^ soil phenolics content were measured. Soil microbial biomass carbon was determined by the method of Vance et al.^34^. Total nitrogen was determined according to the method of Bremner and Mulvaney^35^.

### Statistical analysis

The data of soil parameters was analyzed by one way ANOVA while the data of yield parameters of wheat was analyzed by two way ANOVA. By LSD test means of control and treatment were compared^36^. Pearson correlation was calculated between soil fertility indicators and grain yield of wheat.

### Complies with international, national and/or institutional guidelines

Experimental research and field studies on plants (either cultivated or wild), comply with relevant institutional, national, and international guidelines and legislation. Experimental studies were carried out in accordance with relevant institutional, national or international guidelines or regulation.

### Permissions or licenses

The experiment was started, after taking permission from University of Science and Technology Bannu, Khyber Pakhtunkhwa, Pakistan.

### Identification of the plant material

Before collection, the plant was identified by Dr. Zahid Ullah (Taxonomist), using the standard protocol at the Department of botany, University of Swat, Pakistan.A voucher specimen of this material has been deposited in a publicly available herbarium.

### Ethics approval and consent to participate

We all declare that manuscripts reporting studies do not involve any human participants, human data, or human tissue. So, it is not applicable.

## Results

### Jatropha leaves mulch effect on soil fertility

The effects of Jatropha leaves mulch were investigated on the following indicators of soil fertility status.

### Soil organic matter and nitrogen content

Mulch application significantly improved soil organic matter and nitrogen % as compared to non-mulch control (Table 3). Highest content of organic matter and nitrogen % was found in soil of plots applied with mulch at 5 Mg ha^−1^. Water stress decreased soil organic matter and nitrogen % as compared to irrigated and non-mulch control. However, mulch treated plots exposed to water stress exhibited higher contents of soil organic matter and nitrogen %.

**Table 3:** Effect of *Jatropha curcas* leaves mulch on nitrogen%, organic matter, phenolics content, soil microbial biomass carbon and electrical conductivity under water stress.

Treatments	Organic matter (%)	Nitrogen (%)	Phenolics content (mg gallic acid eq./gram of extract)	Soil microbial biomass carbon (mg/kg)	Electrical conductivity (µS cm^−1^)
Control (unmulched and irrigated)	0.3833 ± 0.01^f^	0.0193 ± 0.00^f^	46.00 ± 0.05^h^	20.350 ± 0.07^d^	249.67 ± 2.60^d^
Mulch (1 Mg ha^−1^) + irrigated	0.4900 ± 0.00^c^	0.0247 ± 0.00^c^	49.10 ± 0.08^g^	23.670 ± 0.16^b^	166.67 ± 10.14^e^
Mulch (3 Mg ha^−1^) + irrigated	0.5200 ± 0.01^b^	0.0263 ± 0.00^b^	50.65 ± 0.21^f^	23.967 ± 0.05^b^	156.67 ± 4.91^e^
Mulch (5 Mg ha^−1^) + irrigated	0.6200 ± 0.01^a^	0.0310 ± 0.00^a^	55.39 ± 0.13^e^	27.747 ± 0.16^a^	132.67 ± 6.49^f^
Water stress (40% soil field capacity)	0.3000 ± 0.01^g^	0.0150 ± 0.00^g^	74.23 ± 0.15^d^	14.190 ± 0.10^f^	384.00 ± 10.21^a^
Mulch (1 Mg ha^−1^) + water stress	0.4100 ± 0.01^e^	0.0207 ± 0.00^e^	106.26 ± 0.06^c^	16.730 ± 0.11^e^	354.00 ± 4.58^b^
Mulch (3 Mg ha^−1^) + water stress	0.4500 ± 0.00^d^	0.0227 ± 0.00^d^	111.20 ± 0.12^b^	21.113 ± 0.08^c^	303.33 ± 9.28^c^
Mulch (5 Mg ha^−1^) + water stress	0.5100 ± 0.01^bc^	0.0257 ± 0.00^bc^	113.19 ± 0.12^a^	23.690 ± 0.08^b^	243.33 ± 8.82^d^

Means with similar English letter are not statistically different. LSD values for nitrogen%: Treatments = 1.223, organic matter: Treatments = 0.0240, phenolics content: Treatments = 0.3738, soil microbial biomass carbon: Treatments = 0.3178, electrical conductivity: Treatments = 22.857.

### Soil phenolics content

The plots supplemented with Jatropha mulch showed considerable increase in soil phenolics content as compared to non-mulch control (Table 3). Maximum phenolics content was found in plots applied with Jatropha mulch at 5 Mg ha^−1^_._ We observed that water stress increased soil phenolics content over irrigated and non-mulch control. However, water stress treated plots applied with mulch showed highest amount of soil phenolics. We noted that soil phenolics content was improved by mulch both under non-stress and water stress conditions.

### Soil microbial biomass carbon

Application of mulch improved soil microbial biomass carbon as compared to non-mulch control (Table 3). Plots treated with 5 Mg ha^−1^ showed highest content of soil microbial biomass carbon. However, water stress significantly decreased soil microbial biomass carbon. The decrease in soil microbial biomass carbon was overcome by application of mulch under water stress conditions.

### Soil electrical conductivity

We examined that electrical conductivity of soil was decreased in plots supplemented with mulch than non-mulch control (Table 3). Water stress treated plots without mulch has high value of electrical conductivity over irrigated and non-mulch control. The mulch application decreased electrical conductivity of soil in plots exposed to water stress. It is stated that application of mulch completely reversed the increase in soil electrical conductivity both in irrigated and water stress treated plots.

### Soil carbonates and bicarbonates

Our results showed that application of mulch significantly decreased soil carbonates and bicarbonates contents as compared to non-mulch control (Table 4). The lowest content of carbonates and bicarbonates was found in plots applied with mulch at 5 Mg ha^−1^. Interestingly water stress treated plots showed significantly higher content of carbonates and bicarbonates as compared to non-mulch and irrigated control. We concluded that Jatropha mulch decreased soil carbonates and bicarbonates content both under normal and water stress conditions.

**Table 4:** Effect of *Jatropha curcas* leaves mulch on carbonates, bicarbonates, calcium and magnesium, chloride ion and total dissolved salts under water stress.

Treatments	Carbonates (meq/L)	Bicarbonates (meq/L)	Ca + Mg (meq/L)	Chloride ion (meq/L)	Total dissolved salts (mg/L)
Control (unmulched and irrigated)	0.9000 ± 0.03^b^	2.1933 ± 0.02^d^	6.0600 ± 0.07^d^	0.9000 ± 0.02^e^	174.77 ± 1.82^d^
Mulch (1 Mg ha^−1^) + irrigated	0.8067 ± 0.03^c^	2.1000 ± 0.02^e^	7.5000 ± 0.12^b^	0.7900 ± 0.02^f^	116.67 ± 7.10^e^
Mulch (3 Mg ha^−1^) + irrigated	0.5033 ± 0.02^e^	2.0267 ± 0.01^f^	8.0533 ± 0.07^a^	0.7000 ± 0.02^g^	109.67 ± 3.44^e^
Mulch (5 Mg ha^−1^) + irrigated	0.2567 ± 0.03^f^	1.6333 ± 0.02^g^	8.5000 ± 0.12^a^	0.5000 ± 0.02^h^	92.87 ± 4.54^f^
Water stress (40% soil field capacity)	0.9767 ± 0.03^a^	4.0000 ± 0.03^a^	5.3333 ± 0.33^e^	2.8933 ± 0.02^a^	268.80 ± 7.15^a^
Mulch (1 Mg ha^−1^) + water stress	0.6500 ± 0.03^d^	3.7033 ± 0.03^b^	6.2267 ± 0.15^cd^	2.7967 ± 0.02^b^	247.80 ± 3.21^b^
Mulch (3 Mg ha^−1^) + water stress	0.5267 ± 0.03^e^	3.0000 ± 0.02^c^	6.5000 ± 0.12^cd^	2.2000 ± 0.02^c^	212.33 ± 6.50^c^
Mulch (5 Mg ha^−1^) + water stress	0.3250 ± 0.02^f^	2.0267 ± 0.01^f^	6.6667 ± 0.09^c^	2.0267 ± 0.01^d^	170.33 ± 6.17^d^

Means with similar English letter are not statistically different. LSD values for carbonates: Treatments = 0.0724, bicarbonates: Treatments = 0.0686, Ca + Mg: Treatments = 0.4645, chloride ion: Treatments = 0.0561, Total dissolved salts: Treatments = 16.000.

### Soil calcium and magnesium

We observed that soil calcium and magnesium content was increased by mulch application as compared to non-mulch control (Table 4). Highest value of calcium and magnesium content was found in plots applied with mulch at 5 Mg ha^−1^. Water stress decreases the content of calcium and magnesium in soil as compared to irrigated and non-mulch control. The decrease in calcium and magnesium content was reversed by mulch application under water stress. Most effective dose of mulch was 5 Mg ha^−1^.

### Soil chloride ions and total dissolved salts

We found that the content of chloride ions and total dissolved salts were lower in plots supplemented with mulch as compared to non-mulch control (Table 4). The plots exposed to water stress and not applied with mulch had higher content of chloride ions and total dissolved salts over irrigated control. The mulch application decreased chloride ions and total dissolved salts in plots exposed to water stress. It is worthy to mention that mulch application at 5 Mg ha^−1^ completely reversed increase in the content of chloride ions and total dissolved salts of water stress treated plots as compared to non-mulch and irrigated control.

### Jatropha leaves mulch effect on wheat yield

#### Grain spike^−1^

Data of treatment means in Table 5 showed useful effect of Jatropha mulch on grain spike^−1^ of wheat. Highest number of grain (16.25%) was recorded for plants supplemented with 5 Mg ha^−1^ of mulch. We noted severe decrease (7.02%) in grain number due to water stress, however, percent decrease in grain number was higher in Pirsabak-2013 (9.77%) than Wadan-2017 (4.81%). It is noted that decreasing effect of water stress on grain number was decreased by Jatropha mulch particularly at 5 Mg ha^−1^ mulch.

**Table 5:** Effect of *Jatropha curcas* leaves mulch on grain per spike and thousand grain weight of wheat under water stress.

Treatments	Grain spike^−1^ (No)			Thousand grain weight (g)		
	V1 (Wadan-2017)	V2 (Pirsabak-2013)	Mean	V1 (Wadan-2017)	V2 (Pirsabak-2013)	Mean
Control (unmulched and irrigated)	55.33 ± 1.45^bc^	44.333 ± 0.33^e^	49.833^c^	51.517 ± 0.82^b−d^	46.850 ± 0.35^gh^	49.183^c^
Mulch (1 Mg ha^−1^) + irrigated	56.667 ± 1.45^b^	45.000 ± 0.58^e^	50.833^c^	52.050 ± 0.65^a−c^	47.050 ± 0.35^f–h^	49.550^bc^
Mulch (3 Mg ha^−1^) + irrigated	57.667 ± 1.45^b^	49.333 ± 1.20^d^	53.500^b^	53.233 ± 0.37^ab^	48.083 ± 0.47^ fg^	50.658^ab^
Mulch (5 Mg ha^−1^) + irrigated	66.000 ± 1.53^a^	53.000 ± 0.58^c^	59.500^a^	53.817 ± 0.38^a^	48.783 ± 0.15^ef^	51.300^a^
Water stress (40% soil field capacity)	52.667 ± 1.45^ cd^	40.000 ± 1.15^f^	46.333^d^	49.983 ± 0.80^de^	42.383 ± 1.05^i^	46.183^d^
Mulch (1 Mg ha^−1^) + water stress	55.667 ± 1.20^bc^	42.667 ± 1.45^ef^	49.167^c^	51.083 ± 0.58^ cd^	42.583 ± 0.50^i^	46.833^d^
Mulch (3 Mg ha^−1^) + water stress	57.333 ± 1.45^b^	43.000 ± 1.00^ef^	50.167^c^	52.767 ± 1.19^a−c^	45.783 ± 0.81^h^	49.275^c^
Mulch (5 Mg ha^−1^) + water stress	58.000 ± 1.53^b^	52.333 ± 1.45^cd^	55.167^b^	53.533 ± 0.15^a^	46.233 ± 0.40^h^	49.883^bc^
Mean	57.417^a^	46.208^b^		52.248^a^	45.969^b^	

Means with similar English letter are not statistically different. LSD values for grain spike^−1^: Treatments = 2.5687, Varieties = 1.2843, T × V = 3.6327: LSD values for thousand grain weight: Treatments = 1.2927, Varieties = 0.6463, T × V = 1.8281.

#### Thousand grains weight

Jatropha mulch at 5 Mg ha^−1^ considerably increased thousand grains weight by 4.13% as compared to well irrigated and non-mulch control (Table 5). Thousand grains weight was decreased (6.10%) by water stress as compared to well irrigated and non-mulch control. On the other hand, use of mulch at 3 and 5 Mg ha^−1^ significantly reversed decrease in thousand grain weights caused by skipped irrigations. Treatment × variety interaction showed that in sensitive variety Pirsabak-2013 percent decrease in thousand grain weights was higher than tolerant Wadan-2017. Both the varieties have better reaction to mulch at 5 Mg ha^−1^.

#### Grain length

Treatment means indicated that under well watered conditions Jatropha mulch at 5 Mg ha^−1^ considerably increased grain length by 7.47% over well irrigated and unmulched control (Table 6). As compared to well irrigated and unmulched control skipped irrigation decreased grain length by 3.79%. However, decrease in seed length was significantly overcome by application of mulch at 3 and 5 Mg ha^−1^. Treatment × variety interaction showed that skipped irrigation highly decreased grain length of sensitive variety Pirssbak-2013 (4.36%) than tolerant Wadan-2017 (3.28%).

**Table 6:** Effect of *Jatropha curcas* leaves mulch on grain length and grain width of wheat under water stress.

Treatments	Grain length (mm)			Grain width (mm)		
	V1 (Wadan-2017)	V2 (Pirsabak-2013)	Mean	V1 (Wadan-2017)	V2 (Pirsabak-2013)	Mean
Control (unmulched and irrigated)	6.8100 ± 0.06^b–d^	6.1167 ± 0.12^fg^	6.4633^cd^	3.3400 ± 0.05^e–h^	3.2967 ± 0.01^f–i^	3.318^de^
Mulch (1 Mg ha^−1^) + irrigated	***6.8967 ± 0.24^a–c^	6.2400 ± 0.20^e–g^	6.5683^c^	3.4067 ± 0.03^b–e^	3.3500 ± 0.03^d–g^	3.3783^cd^
Mulch (3 Mg ha^−1^) + irrigated	6.9300 ± 0.16^a–c^	6.4333 ± 0.17^d–f^	6.6817^bc^	3.4900 ± 0.02^b^	3.4500 ± 0.03^b–d^	3.4700^b^
Mulch (5 Mg ha^−1^) + irrigated	7.2267 ± 0.13^a^	6.7433 ± 0.14^b–d^	6.9850^a^	3.6367 ± 0.00^a^	3.4867 ± 0.01^b^	3.5617^a^
Water stress (40% soil field capacity)	6.5867 ± 0.11^c–e^	5.8500 ± 0.03^g^	6.2183^d^	3.2467 ± 0.03^h–j^	3.1800 ± 0.01^j^	3.2133^f^
Mulch (1 Mg ha^−1^) + water stress	6.6500 ± 0.25^c–e^	6.4200 ± 0.12^d–f^	6.5350^cd^	3.2967 ± 0.03^f–i^	3.2033 ± 0.03^ij^	3.2500^ef^
Mulch (3 Mg ha^−1^) + water stress	6.7700 ± 0.08^b–d^	6.4700 ± 0.04^d–f^	6.6200^bc^	3.3767 ± 0.06^c–f^	3.2667 ± 0.03^g–j^	3.3217^de^
Mulch (5 Mg ha^−1^) + water stress	7.1133 ± 0.06^ab^	6.6900 ± 0.05^cd^	6.9017^ab^	3.4567 ± 0.02^bc^	3.3967 ± 0.08^b–f^	3.4267^bc^
Mean	6.8729^a^	6.3704^b^		3.4062^a^	3.3288^b^	

Means with similar English letter are not statistically different. LSD values grain length: Treatments = 0.2847, Varieties = 0.1423, T × V = 0.4026: LSD values for grain width: Treatments = 0.0729, Varieties = 0.0364, T × V = 0.1031.

It is noted that Wadan-2017 had taller seed length (7.31%) than Pirsabak-2013. Both varieties showed good response to mulch at 5 Mg ha^−1^.

#### Grain width

Treatment means data in Table 6 indicated that Jatropha mulch has favorable effect on grain width of wheat. Highest seed width (3.56 mm) was noted for plants treated with 5 Mg ha^−1^ of mulch over irrigated and unmulched control (3.32 mm). Grain width was significantly decreased (3.16%) by skipped irrigation. However, as compare to Wadan-2017 skipped irrigation highly decreased grain width in Pirsabak-2013. It is noted that skipped irrigation effects was minimized by Jatropha mulch. As compared to other mulch treatments 5 Mg ha^−1^ mulch was more effective.

#### Spike weight

Application of mulch at 3 and 5 Mg ha^−1^ considerably increased spike weight (13.36% and 17.33%) respectively (Table 7). Major reduction (14.74%) occurred in spike weight due to skipped irrigation. Percent decrease in spike weight was higher in Pirsabak-2013 (17.53%) as compared to Wadan-2017 (12.42%) due to skipped irrigation. However, skipped irrigation does not significantly effects the spike weight of plants treated with Jatropha mulch. It is noted that application of mulch at 5 Mg ha^−1^ significantly increase spike weight of wheat both under skipped irrigated and well irrigated conditions.

**Table 7:** Effect of *Jatropha curcas* leaves mulch on spike weight and awn length of wheat under water stress.

Treatments	Spike weight (g)			Awn length (cm)		
	V1 (Wadan-2017)	V2 (Pirsabak-2013)	Mean	V1 (Wadan-2017)	V2 (Pirsabak-2013)	Mean
Control (unmulched and irrigated)	3.2467 ± 0.24^cd^	2.7000 ± 0.03^ef^	2.9733^cd^	6.3333 ± 0.44^b–e^	6.3333 ± 0.33^b–e^	6.3333^bc^
Mulch (1 Mg ha^−1^) + irrigated	3.3433 ± 0.03^bc^	2.9000 ± 0.03^cf^	3.1217^bc^	6.6667 ± 0.33^a–c^	6.5000 ± 0.29^a–d^	6.5833^ab^
Mulch (3 Mg ha^−1^) + irrigated	3.7867 ± 0.01^ab^	3.0767 ± 0.01^c–e^	3.4317^ab^	6.8333 ± 0.17^ab^	6.6667 ± 0.17^a–c^	6.7500^ab^
Mulch (5 Mg ha^−1^) + irrigated	3.9433 ± 0.02^a^	3.2500 ± 0.02^cd^	3.5967^a^	7.0000 ± 0.00^a^	6.8333 ± 0.17^ab^	6.9167^a^
Water stress (40% soil field capacity)	2.8433 ± 0.24^d–f^	2.2267 ± 0.01^g^	2.5350^e^	6.0000 ± 0.00^d–f^	5.5000 ± 0.03^f^	5.7500^d^
Mulch (1 Mg ha^−1^) + water stress	2.9633 ± 0.09^c–f^	2.5500 ± 0.13^fg^	2.7567^de^	6.1667 ± 0.17^c–e^	5.9000 ± 0.03^ef^	6.0333^cd^
Mulch (3 Mg ha^−1^) + water stress	3.0033 ± 0.30^c–f^	2.8900 ± 0.09^c–f^	2.9467^cd^	6.6667 ± 0.17^a–c^	6.4000 ± 0.06^b–e^	6.5333^ab^
Mulch (5 Mg ha^−1^) + water stress	3.7133 ± 0.36^ab^	2.9733 ± 0.29^c–f^	3.3433^ab^	6.8333 ± 0.17^ab^	6.5333 ± 0.03^a–d^	6.6833^ab^
Mean	3.3554^a^	2.8208^b^		6.5625^a^	6.3333^b^	

Means with similar English letter are not statistically different. LSD values spike weight: Treatments = 0.3216, Varieties = 0.1608, T × V = 0.4548: LSD values for awn length: Treatments = 0.4177, Varieties = 0.2088, T × V = 0.5907.

#### Awn length

Data of treatment means in Table 7 showed that as compared to well irrigated and unmulched control awn length of wheat spike was significantly increased (8.43%) by Jatropha mulch at 5 Mg ha^−1^ under well irrigated conditions. Awn length was decreased (9.21%) by skipped irrigation than unmulched control and well irrigated. Moreover, decrease in awn length was significantly overcome by 3 and 5 Mg ha^−1^ mulch. Decrease in awn length was higher in sensitive variety Pirssbak-2013 (13.15%) than tolerant Wadan-2017 (5.26%) due to skipped irrigation. It is noted that variety Wadan-2017 has higher awn length than Pirsabak-2013.

#### Straw weight

Treatment means data showed that mulch at 3 and 5 Mg ha^−1^ respectively increase straw weight (35.01% and 45.79%) in non stressed groups (Table 8). Skipped irrigation significantly decrease (23.53%) straw weight. Straw weight was considerably decreased in Pirsabak-2013 (29.29%) as compared to Wadan-2017 (18.89%) by skipped irrigation. Moreover, straw weight treated with Jatropha mulch was not affected by skipped irrigation. It is examined that both under skipped irrigated and well irrigated conditions straw weight of wheat was increased by application of mulch at 5 Mg ha^−1^. Wadan-2017 had high straw weight (20.05%) than Pirsabak-2013.

**Table 8:** Effect of *Jatropha curcas* leaves mulch on straw weight and grain yield of wheat under water stress.

Treatments	Straw weight (kg ha^−1^)			Grain yield (kg ha^−1^)		
	V1 (Wadan-2017)	V2 (Pirsabak-2013)	Mean	V1 (Wadan-2017)	V2 (Pirsabak-2013)	Mean
Control (unmulched and irrigated)	12.000 ± 0.289^de^	9.900 ± 0.058^gh^	10.950^e^	4200.0 ± 5.8^ef^	3883.3 ± 44.1^h^	4041.7^e^
Mulch (1 Mg ha^−1^) + irrigated	18.500 ± 0.289^b^	10.500 ± 0.289^f–h^	14.500^c^	4750.0 ± 28.9^d^	4050.0 ± 28.9^f–h^	4400.0^d^
Mulch (3 Mg ha^−1^) + irrigated	22.000 ± 0.764^a^	11.683 ± 0.428^d–f^	16.842^b^	5483.3 ± 8.8^ab^	4950.0 ± 28.9^c^	5216.7^a^
Mulch (5 Mg ha^−1^) + irrigated	22.940 ± 0.266^a^	17.450 ± 0.770^b^	20.195^a^	5620.0 ± 11.5^a^	5083.3 ± 44.1^c^	5351.7^a^
Water stress (40% soil field capacity)	9.747 ± 0.087^h^	7.000 ± 0.289^i^	8.373^f^	4000.0 ± 11.5^gh^	3600.0 ± 28.9^i^	3800.0^f^
Mulch (1 Mg ha^−1^) + water stress	11.043 ± 0.275^e–g^	9.500 ± 0.173^h^	10.272^e^	4130.0 ± 43.6^fg^	4100.0 ± 28.9^fg^	4115.0^e^
Mulch (3 Mg ha^−1^) + water stress	11.260 ± 0.167^ef^	14.667 ± 0.167^c^	12.963^d^	4996.7 ± 57.8^c^	4350.0 ± 28.9^e^	4673.3^c^
Mulch (5 Mg ha^−1^) + water stress	12.757 ± 0.884^d^	15.350 ± 0.176^c^	14.053^c^	5400.0 ± 28.9^b^	4700.0 ± 115.5^d^	5050.0^b^
Mean	15.031^a^	12.006^b^		4822.5^a^	4339.6^b^	

Means with similar English letter are not statistically different. LSD values straw weight: Treatments = 0.8442, Varieties = 0.4221, T × V = 1.1939: LSD values for grain yield: Treatments = 135.03, Varieties = 67.515, T × V = 190.96.

#### Grain yield

Table 8 indicated that 5 Mg ha^−1^ Jatropha mulch significantly increased (24.48%) grain yield of wheat as compared to unmulched and irrigated control. Skipped irrigation considerably decreased (5.98%) grain yield of wheat as compared to unmulched and irrigated control. The percent decrease due to skipped irrigation in grain yield of wheat was higher in Pirsabak-2013 (7.30%) compared to Wadan-2017 (4.7%). However, the decrease in grain yield of wheat was overcome by leaves mulch of Jatropha. Comparably Wadan-2017 had high grain yield (10.01%) than Pirsabak-2013.

#### Pearson’s correlation coefficient (r)

**Figure 1:**
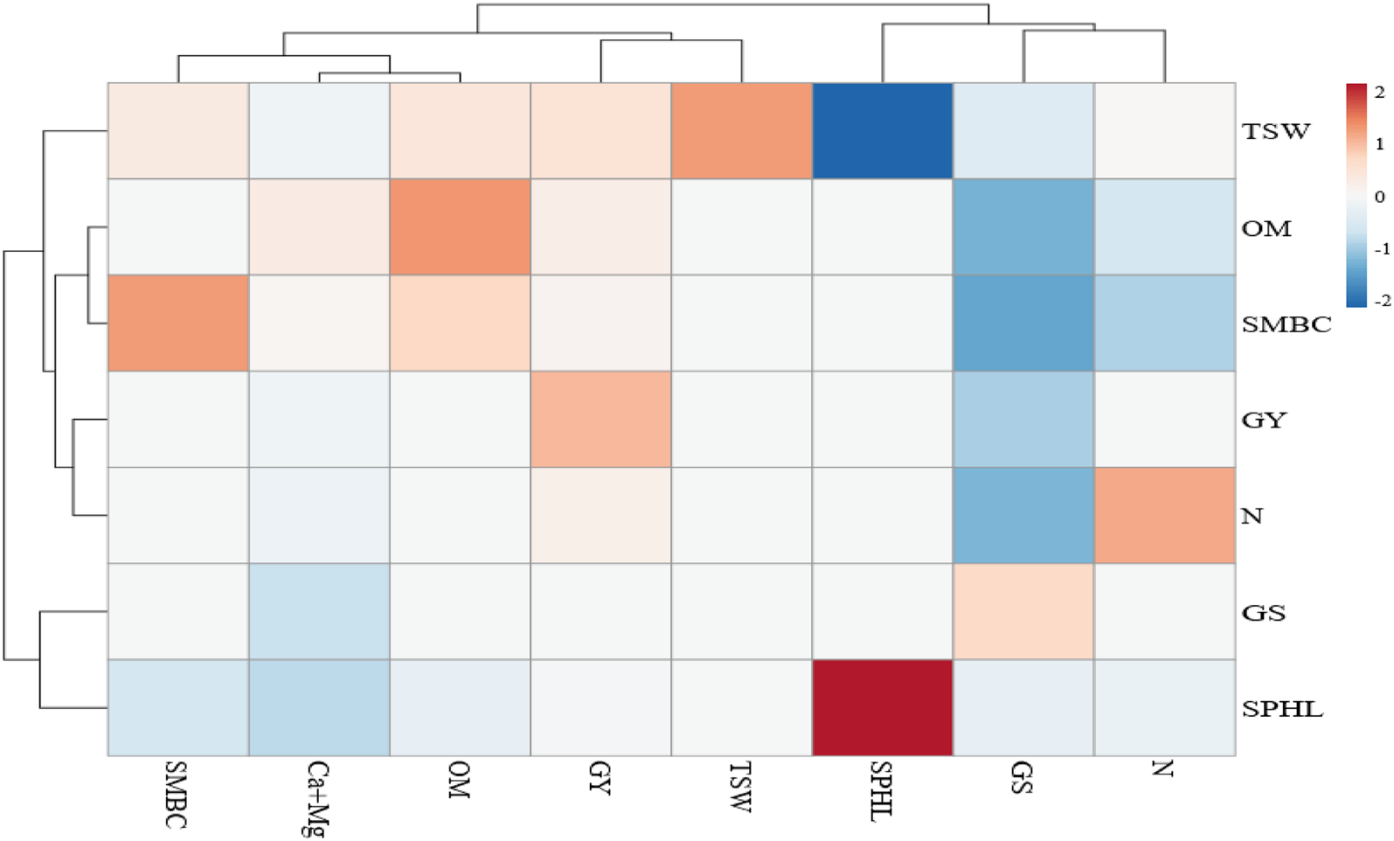
Pearson’s Correlation Coefficient (r) shown by heatmap for the soil fertility indicators and yield related traits of wheat under water stressed condition. Blue color is indicator of negative correlation whereas, peach to maroon colors indicate positive correlation. The color allotted to a specific point in the heat map is indicator of the strength of correlation between two specific traits.

Heat map analysis (Fig. 1) revealed that grain yield was significantly positive correlated with soil N content (r = 0.9895), soil organic matter (r = 0.9951), soil phenolics (r = 0.9294), soil microbial biomass carbon (r = 0.9955), thousand grains weight (r = 0.9946), and soil Ca + Mg (r = 0.9961). Soil N content was significantly positive correlated with organic matter (r = 0.9914), soil phenolics (r = 0.9026), soil microbial biomass carbon (r = 0.9897), and thousand seed weight (r = 0.9770). Soil organic matter content was significantly positive correlated with soil phenolics (r = 0.9101), soil microbial biomass carbon (r = 0.9979), and thousand seeds weight (r = 0.9846). Soil microbial biomass carbon was considerably positively correlated with thousand seed weight (r = 0.98610).

## Discussion

Jatropha mulch showed positive effects on soil organic matter content. A soil organic matter contains organic compounds released from dead and decaying of living organisms^37^ and improves soil health^38^. Soil organic matter improves water holding capacity of soil and this is the major reason that organic mulches minimize water stress effects on the establishment of crops^39^. Organic matter resulting from microbial degradation of mulch releases nutrients to the soil and thus improving soil fertility^40^.

We recorded improvements in soil total nitrogen (%) due to Jatropha mulch application. Mulching improves nutrients cycle in cultivated lands^41^ leading to better establishment of crops^42^. Nitrogen content was high in the plots supplemented with maize straw mulch^43^. Studies have shown impact of mulches on soil N and C pools^44^. Organic mulching materials of oak (*Quercus fabri*), cogon grass (*Imperata cylindrica*), bracken fern (*Pteridium aquilinum*) and Chinese coriaria (*Coriaria nepalensis*) increased soil nitrogen content which was directly proportional to the decaying rate and nutrients content of the mulching material^45^.

We reported that water stress decreased soil microbial biomass in non-mulch plots; however, Jatropha mulch improved soil microbial biomass both under non-stress and water stress conditions. Microbial biomass shows considerable response to the climatic conditions and soil micro environment. Changing pattern of rainfall and global warming effects the reproduction and growth of soil microbes^46–48^. Therefore, in soil ecosystem degradation, microbial biomass carbon content acts as an early warning indicators^49^. Many researchers have reported that with the increase of drought stress soil microbial activity becomes damaged^50–52^ and decreased up to 39%^53^. Our studies indicated that Jatropha leaves mulch improved soil microbial biomass which assisted in better establishment of wheat crop under low soil moisture availability.

Phenolics concentration was higher in plots supplemented with Jatropha mulch. Studies of Stoklosa et al.^54^ showed that phenolics concentration was higher in soil provided with rye and oat mulch. Higher content of phenolics in plots applied with mulch may be because that Jatropha leaves contained a reasonable amount of phenolics. Although phenolics are highly reactive having phytotoxicity yet they are degraded either by soil microbes or by oxidation limiting their allelopathic potential in mulching trials^55^.

We noted a severe decrease in yield related traits of wheat due to imposed water stress. Water stress considerably minimized the yield of wheat^56^. This reduction in grain yield of wheat under water stress may be due to leaf senescence acceleration, degeneration of photosynthesis and sink restrictions^56^. In our studies Jatropha mulch minimized water stress negative impact on grain yield of wheat. Furthermore, decrease in grain number per spike was due to reduction in spikelets number per spike of wheat^57^. Studies have shown that black plastic mulch and rice straw mulch significantly increased straw yield, thousand seed weight and seed yield of wheat respectively^29^. Deng et al.^58^ have also described the benefits of mulching on yield of wheat. Water stress reduced the grain and straw yield as compared to well watered treatments. Application of Jatropha mulch on grain yield may be majorly due to its improving effects on soil organic matter and soil nitrogen content as reported earlier^59^. Our results are also in confirmation with those of^60^ that dual transparent plastic film + straw mulching and dual black plastic film + straw mulching considerably improved soil temperature, biomass, soil water storage potential, total nitrogen, soil organic carbon and grain yield of wheat. Arbuscular mycorrhizal fungi significantly increased water use efficiency, soil microbial biomass carbon/nitrogen ratio and crop productivity of non-irrigated wheat^61^.

## Conclusion

Application of Jatropha mulch improved soil microbial activity, total N, organic matter and phenolics content as compared to non-mulch and irrigated control. Water stress restricted microbial biomass production, decreased soil nitrogen content and grain yield of wheat. The application of *Jatropha curcas* leaves mulch reversed negative effects of water stress on grain yield and soil fertility status. Findings of our research are novel in the sense that Jatropha leaves mulch was beneficial on soil microbial biomass and soil phenolics which might contribute in the reduction of disease attacks on wheat crop. Moreover, phenolics present in mulch have made a way of diffusion into the soil which might have assisted in the water stress resistance of wheat varieties. It was found that both the wheat varieties showed similar response to Jatropha mulch application irrespective of their tolerance level to water deficit stress.

## Data Availability

The datasets generated and/or analysed during the current study are not publicly available, but are available from the corresponding author on reasonable request.
